# Interrelationship between muscle fitness in childhood and bone mineral density in adulthood: mediation analysis of muscle fitness in adulthood

**DOI:** 10.1186/s12889-023-15545-7

**Published:** 2023-04-04

**Authors:** Cynthia Correa Lopes Barbosa, Julio Cesar da Costa, Catiana Leila Possamai Romanzini, Mariana Biagi Batista, Gabriela Blasquez-Shigaki, Rômulo Araújo Fernandes, Diogo V. Martinho, Tomás Oliveira, Luís P. Ribeiro, Manuel João Coelho-e-Silva, Enio Ricardo Vaz Ronque

**Affiliations:** 1 Laboratory of Physical Activity and Health, Center of Physical Education and Sports, Londrina State University - UEL, Highway Celso Garcia Cid, Km 380, University Campus, P.O. box 6001, 86051-990 Londrina, Paraná Brazil; 2 Department of Humanities, Federal Technological University of Paraná - UTFPR, Apucarana, Paraná Brazil; 3 Federal University of Mato Grosso do Sul – UFMS, Mato Grosso do Sul, Campo Grande, Brazil; 4 Department of Physical Education, Exercise Research Laboratory (LIVE), Faculdade de Ciências e Tecnologia, State São Paulo University – UNESP, Presidente Prudente, São Paulo, Brazil; 5 Faculty of Sports Sciences and Physical Education, University of Coimbra, Coimbra, Portugal; 6 University of Algarve, Faro, Portugal

**Keywords:** Physical fitness, Muscle strength, Bone health, Growth

## Abstract

**Background:**

This study was aimed to examine the relationship between muscular fitness indicators in childhood and areal bone mineral density (aBMD) in adulthood and to verify whether the relationship is mediated by performance on muscular fitness indicators in adulthood.

**Methods:**

A sample of 138 healthy adults (69 males; 22.3 years) were followed after a previous assessment at the age of 7–10 years. Stature, body mass and muscular fitness indicators (handgrip strength, standing long jump and sit-ups tests) were assessed in childhood and adulthood. Additionally, total body, upper limbs, lower limbs, right femoral neck and lumbar spine aBMD was assessed in adulthood using dual X-ray absorptiometry. Analysis included descriptive statistics; t-test or Mann-Whitney U-test for comparison between males and females, multiple linear regression for the prediction aBMD from muscular fitness indicators in childhood, mediation analysis of the respective muscular fitness indicators in adulthood and the relationship between muscular fitness indicators in childhood and aBMD.

**Results:**

Males were stronger compared to females regarding muscular fitness indicators in childhood and adulthood, and presented higher mean values for aBMD in adulthood, except for lumbar spine (p < 0.05). Regression analysis revealed that some muscular fitness indicators in childhood showed significant positive relationship with bone health indicators in adulthood, such as: handgrip strength and total body aBMD (β = 0.005; R^2^ = 0.35; p = 0.040) and upper limbs aBMD (β = 0.005; R^2^ = 0.55; p = 0.019); and sit-ups test was a significant predictors of lumbar spine BMD (β = 0.003; R^2^ = 0.06; p = 0.039). Mediation analysis pointed out the following: adulthood handgrip strength mediated relationships between childhood handgrip strength and total aBMD (indirect effect (IE) = 0.0025; 95%CI = 0.0005–0.0048), and upper limbs aBMD (IE = 0.0040; 95%CI = 0.0017–0.0069).

**Conclusions:**

Muscular fitness indicators in childhood showed significant relationship with bone health indicators in adulthood and the sit-ups test in childhood had direct effect on lumbar spine aBMD in adulthood. Adulthood handgrip strength mediated the relationship between childhood handgrip strength and total body and upper limb aBMD, pointing out that muscular fitness in childhood may be a aBMD determinant in adulthood, especially when higher muscle fitness performance is maintained in adulthood.

## Introduction

Peak bone mass (PBM) is reached in early adulthood and is interpreted as the achievement of the highest expression of bone tissue [1]. It is considered a relevant indicator in prediction of osteoporosis and fractures in the course of aging [2, 3]. The literature suggests that an increment of 10% in PBM tends to delay the onset of osteoporosis by 13 years [4]. In addition, epidemiological evidences [5] concluded that this increase of 10% in PBM during the first decades of life is associated to a decrement of 50% in the risk of fracture among elderly women. By inference, optimization of the bone mass gain during growth seems central to mitigate the consequences of the physiological loss with aging.

Several factors contribute to PBM such as genetics, bone status during childhood, endocrine regulators, interaction of bone tissue with other tissues, lifestyle factors, chronic diseases during childhood, and others [6–8]. Meantime, the trajectory of bone mass gains is similar to that of linear growth and believed to be sensitive to modifiable or non-modifiable determinants during this period [9]. There is evidence of the effect of physical activity [7] and improved muscular fitness on bone mass [10]. Bones are exposed to muscular action, stronger muscles exert more tension on bones and consequent mechanical adaptations. The mechanical adaptations of bones according to what type of exercise they are submitted to are explained by mechanostatic [11], mechanosensation and transduction theories [12].

Positive relationship between muscle fitness and bone health indicators between children and adolescents aged 8–18 years, has been previously demonstrated in cross-sectional studies [13, 14], but evidence that muscular fitness in childhood and adolescence is a determinant of bone health in adulthood is still limited [15]. Recently, García-Hermoso et al. [16] performed a meta-analysis to conclude for a moderate effect of muscle fitness during childhood and adolescence on follow-up studies assessing areal bone mineral density (aBMD). Although evidence about the impact of mechanical stress through muscle action on bone modulation [17], it is not clear whether the advantages obtained are maintained when the physical stimulus ceases [18, 19]. A few studies observed the maintenance of benefits from exposure to mechanical loads even years after physical activity has ceased [20, 21]. In contrast, other studies showed that gains were not maintained after activity cessation or reduction [22, 23].

Adult males and females who had been physically active during adolescence demonstrated bone mineral content 8 to 10% higher compared to inactive or moderately active peers during adolescence, and also had higher physical activity scores in adulthood [24]. Meantime, the association between muscle fitness indicators (MFI) in circumpubertal years and bone strength variables in adulthood could be attenuated after the model was adjusted for the performance of muscle fitness indicators in adulthood [25]. Thus, it is of interest to identify the direct, indirect or mediated effects by performance in MFI in adulthood on the relationship between muscle fitness in childhood and aBMD in adulthood. The current study is aimed to examine the relation-ship between MFI in childhood and different aBMD regions of interest in adulthood and to verify whether this relationship is mediated by performance of MFI in adulthood.

## Methods

### Procedures and ethics committee

This prospective study was initially designed to examine growth, maturation and physical fitness in schoolchildren aged 7 to 10 years recruited from a private school in Londrina (Paraná, Brazil), with a mixed longitudinal design with four birth cohorts (1992, 1993, 1994, 1995) followed annually from 2002 to 2006 (initial moment research approved by ethics committee of Campinas State process CEP N 249/2002, July 16, 2002, and longitudinal data by ethics committee of Londrina State University process CEP N 024/03, April 01, 2003). Criteria of sampling were an α of 95%, a statistical power of 80%, and an error of 5% as previously described [26]. Parents or legal guardians of 1052 children signed an informed consent, and participants were informed that their participation was voluntary. Data were collected during Physical Education requiring three visits of the research team to elementary school within two weeks in all follow-up years. Participants of the preceding sample were contacted 15 years later to be reassessed as part of the “Physical fitness and practice of sports in childhood and adolescence and behavioral risk factors in adulthood” (ethics committee of Londrina State University approved the research: process N 1.340.735, November 27, 2015). The baseline and follow-up have been previously described [27].

### Sample

**Fig. 1:**
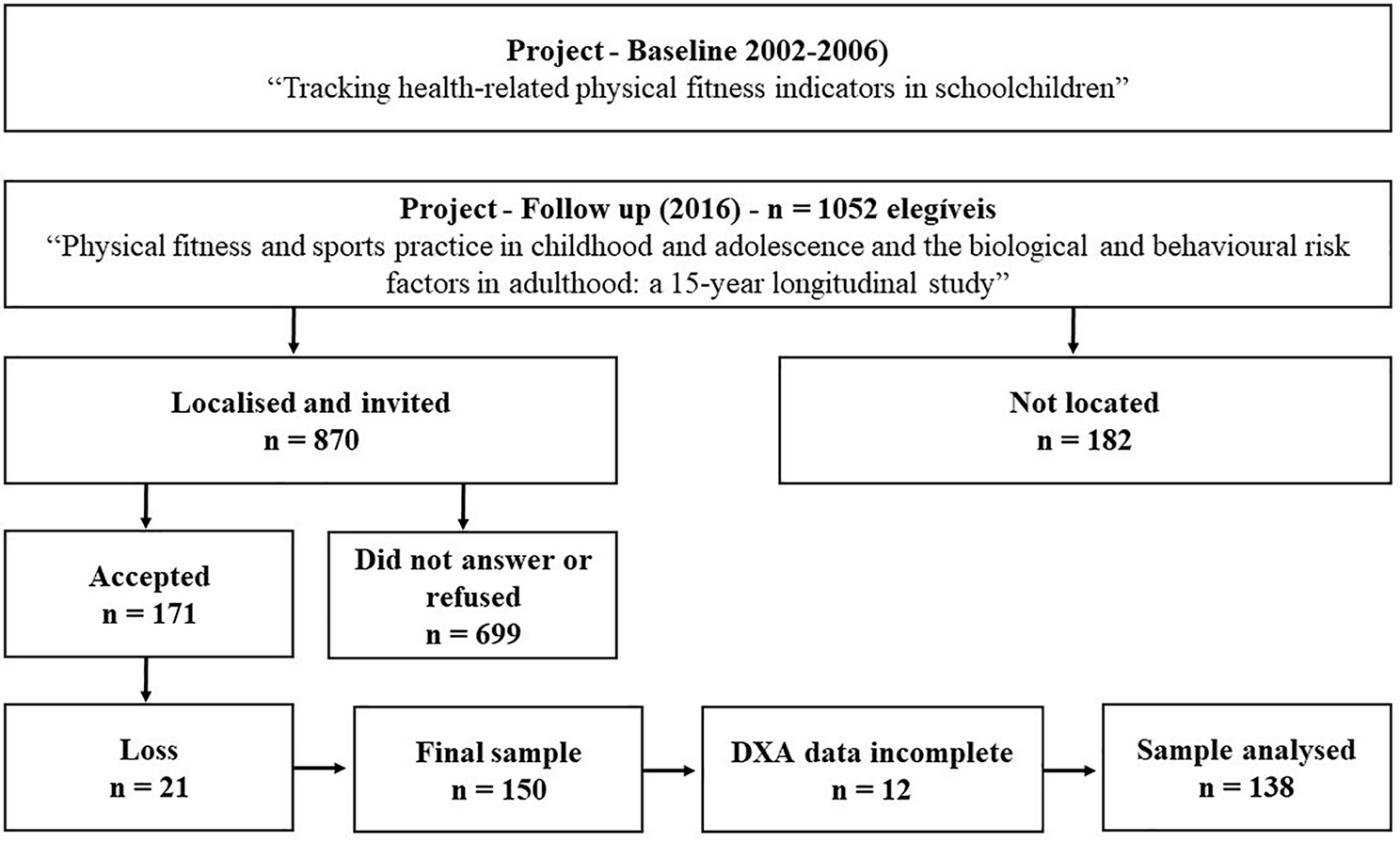
Sample selection

Inclusion criteria for the current study were: (i) not being injured or physically limited (as, for example, asthma); (ii) have at least one baseline measure for fitness tests to determined MFI were assessed; (iii) completed the same muscular fitness indcators battery as adults in addition to dual energy x-ray absorptiometry. Exclusion criteria were: participants under frequent use of medication to treat any disease that could interfere with the study variables (Fig. 1).

A total of 138 healthy young adults aged 18–25 years were eligible to participate in the study (50% males). Examination of dropouts compared to children who were assessed at baseline and follow-up is summarized in Table 1. Differences between children who participated as adults and those who were evaluated only at baseline did not indicate significant differences in variables, except for the sit-up test in males (p = 0.02).

**Table 1:** Characteristics at childhood for those included in this analysis and dropped out separately for males and females

		Males							Females			
		Follow-up (n = 69)			Dropout (n = 424)	p			Follow-up (n = 69)		Dropout (n = 490)	p
Chronological age	years	9.0 ± 1.6			9.2 ± 1.8	0.47			9.3 ± 1.3		9.0 ± 1.7	0.19
Body mass	kg	32.1 ± 9.5			33.7 ± 10.3	0.22			33.6 ± 8.2		34.1 ± 10.7	0.75
Stature	cm	134.8 ± 11.6			135.3 ± 11.5	0.71			136.0 ± 9.3		135.2 ± 11.4	0.28
Body mass index	kg/m^2^	17.3 ± 2.8			18.0 ± 3.2	0.07			17.97 ± 2.6		18.3 ± 3.2	0.47
Handgrip strength	kgf	17.3 ± 4.8			17.5 ± 5.4	0.75			19.2 ± 4.9		18.9 ± 6.3	0.61
Standing long jump	cm	128.8 ± 19.4			122.9 ± 23.9	0.05			139.7 ± 22.0		135.6 ± 20.3	0.14
Sit-ups	rep	29.4 ± 8.1			28.8 ± 9.3	0.63			34.1 ± 9.1		31.5 ± 8.8	0.03*

Note: * = P < 0.05

### Anthropometry

Anthropometry was assessed according to procedures described by Gordon et al. [28]. Body mass was measured on a digital platform scale with precision of 0.05 kg. Harpenden portable stadiometer with 0.1 cm precision was used to measure height. Subsequently, body mass index (BMI) was calculated and expressed in kg per m^2^. Childhood BMI z-score was calculated using reference values from the CDC Growth Charts [29] and was used as an adjustment variable in the statistical analyses.

### Muscular fitness indicators (MFI)

Muscle fitness is understood as the capacity to perform work against resistance and involves maximum isometric or dynamic strength, isokinetics, muscular endurance and power [10]. The hand grip strength test (HS), strength measure, was completed according to procedures described by Soares and Sessa [30], using Jamar Hydraulic Dynamometer (Sammons and Preston Scientific Industries Inc.) with precision of 1 kilogram force (kgf). Three measurements were performed and the best score was retained for analysis. Standing long jump test (SLJ), explosive strength indicator, corresponded to the maximal horizontal jumping performance performed from a starting position with participant with parallel feet. Three attempts were performed with the best scores in cm retained for analysis [30]. Sit-ups test, endurance strength indicator, required a mat and a stopwatch. With participants in dorsal decubitus, hips and knees flexed, feet soles facing the ground, arms crossing the thorax, hands supported on shoulders, the evaluator was holding the feet of participants who were instructed to perform the maximum number of trunk elevation including a contact of the forearms with the thighs and return to the initial position, the test was performed only once for a period of 60 seconds and the total number of repetitions was used in the analyses [31]. For the analysis, the total number of repetitions performed on a single trial was recorded.

In addition to the performance in each indicator of muscular fitness during the childhood period, an index called childhood muscular fitness z-Score (CMF z-Score) was calculated by adding the standardized z-score value (individual value - mean / standard deviation) of the performance in each indicator of muscular fitness (HS + SLJ + Sit-ups).

Regarding the quality control of observed data, the muscular fitness indicators of 20 adults randomly selected, after an interval of 7 days, were analyzed. Intraclass correlation coefficients for intra-observer reliability were: HS (ICC = 0.98), SLJ (ICC = 0.98) and sit-ups (ICC = 0.90).

### Dual energy X-ray absorptiometry (DXA)

Participants were positioned on the table in supine position with the body aligned along with the central axis. A single certified technician completed the scans using DXA (Lunar DPX-MD+, GE Lunar Corporation, 726 Heartland Trail, Madison, WI 53717 − 1915 USA). Data were obtained using the software recommended by the manufacturer. Scans allowed calculations for aBMD of total body, lumbar spine (L1-L4), upper limbs, lower limbs, right femoral neck. The equipment was previously calibrated according to manufacturer. Full body scan was performed with participants in supine position and aligned, holding still for approximately 15 to 20 minutes. For the lumbar region, individuals were also positioned in dorsal decubitus, with legs placed on a block forming a 90-degree angle in relation to the table, with the intention of straightening the lumbar spine. For the proximal femur examination, keeping the patient positioned in dorsal decubitus, a triangular support was used to immobilize the lower limbs after internal rotation and adequate positioning of the femur, in order to capture the femoral neck region of interest.

### Data analysis

Descriptive statistics of the sample were summarized in Table 2, separately for baseline and follow-up and sex. Comparisons between males and females were determined using independent t-test at baseline and follow-up. Linear regression using the enter method was used to analyze the relationship between MFI measured in childhood (baseline) and aBMD of different regions of interest measured in adulthood (follow-up). Regression analyses were adjusted for sex, chronological age, and childhood BMI z-score. Mediation analysis was performed on the MFI in childhood that showed statistically significant relationship with bone health indicators (BHI), considering the respective MFI in adulthood as the mediator variable. Mediation analysis followed the principles of Baron and Kenny [32] using the PROCESS 3.0 macro by Andrew F. Hayes, where “a” reflected the relationship between independent variable and the proposed mediator variable, “b” was the effect of the mediator variable on the dependent variable, partializing the effect of the independent variable, “c’” represented the direct impact of the independent variable on the dependent variable, and, “c’” represented the total effect of the independent variable on the dependent variable. The indirect effect is the product of “a” and “b” and quantifies the effect of the independent variable on the dependent variable through the mediator variable. Mediation assumptions were confirmed, the confidence interval of the indirect effect was estimated by the bootstrapping technique (5000, resampling), and unstandardized parameters were used to describe Betas. The mediation proportion was estimated by calculating 1 - (direct effect/total effect). Data were analyzed using SPSS version 25.0. The significance level adopted was 5%.

**Table 2:** Descriptive statistics and comparisons between males and females at baseline and adulthood

				Baseline					Follow-up			
		Females (n = 69)			Males (n = 69)	p			Females (n = 69)		Males (n = 69)	p
Chronological age	years	9.0 ± 1.6			9.3 ± 1.3	0.25			22.2 ± 1.7		22.4 ± 1.7	0.587
Body mass	kg	32.1 ± 9.5			33.6 ± 8.2	0.29			60.3 ± 10.7		76.1 ± 10.6	< 0.001
Stature	cm	134.8 ± 11.6			136.0 ± 9.3	0.50			164.6 ± 6.7		176.5 ± 6.0	< 0.001
Body mass index	kg/m^2^	17.29 ± 2.8			17.97 ± 2.6	0.15			22.21 ± 3.4		24.40 ± 2.9	< 0.001
Hand grip strength	kgf	17.3 ± 4.9			19.2 ± 4.7	< 0.01			28.7 ± 5.5		49.7 ± 8.7	< 0.001
Standing long jump	cm	128.8 ± 19.4			139.7 ± 22.0	< 0.01			152.7 ± 17.7		206.2 ± 24.4	< 0.001
Sit-ups	rep	29.4 ± 8.1			34.1 ± 9.1	< 0.01			37.4 ± 10.2		47.6 ± 9.8	< 0.001
Bone mineral density												
Total body	g/cm^2^								1.167 ± 0.074		1.269 ± 0.091	< 0.001
Lumbar spine	g/cm^2^								1.174 ± 0.123		1.205 ± 0.135	0.162
Upper limbs	g/cm^2^								0.795 ± 0.049		0.945 ± 0.092	< 0.001
Lower limbs	g/cm^2^								1.203 ± 0.095		1.430 ± 0.122	< 0.001
Right femoral neck	g/cm^2^								1.038 ± 0.124		1.165 ± 0.184	< 0.001

## Results

Table 2 summarize the mean and standard deviation of males and females separately at the baseline and follow-up. At baseline, although boys and girls did not differ in terms of body size given by stature, body mass and BMI, significant differences were noted for the MFI (HS, p < 0.01; SLJ, p < 0.01; sit-ups, p < 0.01). Meantime, among the adult sample, males were heavier (p < 0.001), taller (p < 0.001), stronger (HS, p < 0.001; SLJ, p < 0.001; sit-ups, p < 0.001). Regarding aBMD that was uniquely assessed among adults, sex differences were significant for total body (p < 0.001), upper limbs (p < 0.001), lower limbs (p < 0.001), right femoral neck (p < 0.001).

The association between MFI in childhood and aBMD of the different regions of interest in adulthood and significant values were obtained (Table 3). In the multiple linear regression procedure, adjustments for sex, age, and childhood BMI z-score were considered. Muscular fitness in childhood HS was significant predictors of total body aBMD (p = 0.040), trunk aBMD (p = 0.017) and upper limbs aBMD (p = 0.019). The sit-ups test was significant predictors of lumbar spine aBMD (p = 0.039) and trunk aBMD (p = 0.036), and the CMF z-Score was significant predictors of trunk aBMD (p = 0.040). The other relationships were not statistically significant.

**Table 3:** Association between muscular fitness indicators in childhood and bone mineral density of different regions of interest in adulthood

Variables		Total Body aBMD (g/cm^2^)				Trunk aBMD (g/cm^2^)				Arm aBMD (g/cm^2^)				Lumbar spine aBMD (g/cm^2^)		
		β	R^2^	p	-	β	R^2^	p	-	β	R^2^	p	-	β	R^2^	p
Childhood HS (kgf)		0.005	0.35	0.040		0.006	0.24	0.017		0.002	0.55	0.019		-	-	-
Childhood sit-ups (rep)		-	-	-		0.002	0.23	0.036		-	-	-		0.003	0.06	0.039
CMF z-Score		-	-	-		0.008	0.23	0.040		-	-	-		-	-	-

Note: HS = Dominant handgrip strength; aBMD = Areal bone mineral density; CMF z-Score = Childhood Muscular Fitness z-Score; Model adjusted for gender, childhood chronological age, and childhood BMI Z-score; Significance level at P < 0.05

**Fig. 2:**
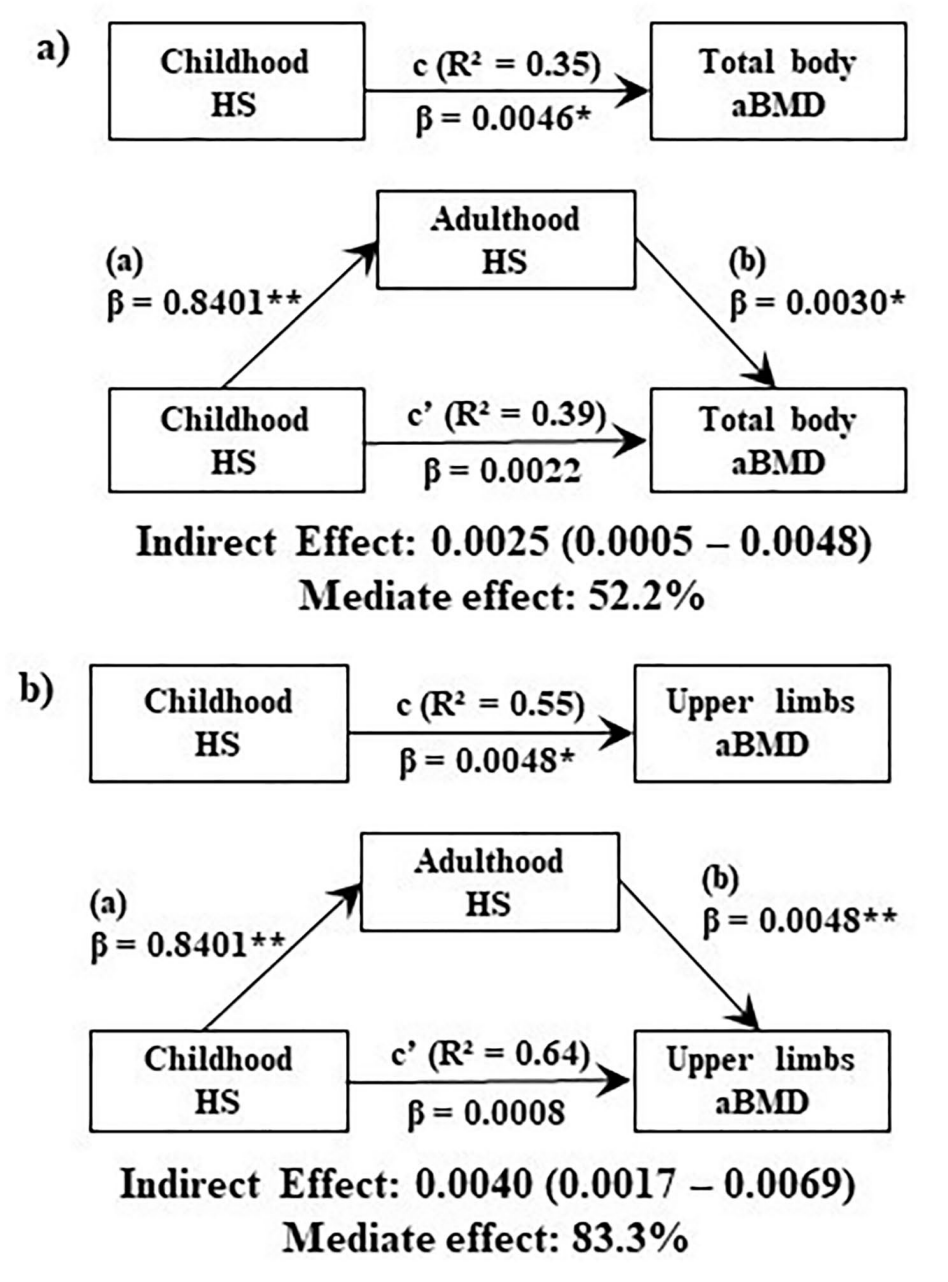
Mediation models for muscular fitness indicators in adulthood on the relationship between muscular fitness indicators in childhood and BMD of different regions of interest in adulthood, non-standard parameters **Note**: HS = Handgrip strength; aBMD = Areal bone mineral density; a = Effect of the independent variable on the pro-posed mediating variable; b = Effect of the variable proposed as mediator on the dependent variable with partial effect of the independent variable; c’ = Direct effect of the independent variable on the dependent variable; c = Total effect of the independent variable on the dependent variable. Adjusted model by sex, childhood chronological age, and childhood BMI z-score; * = p < 0.05; ** = p < 0.01

Subsequently to this identification, the respective MFI measured at follow-up, i.e., in early adulthood, was evaluated as mediators in the relationship between MFI in childhood and BHI in adulthood. The mediation analyses can be seen in Fig. 2 only in the models that attended the statistical assumptions.

Considering the coefficients of regression equations in the mediation analysis identified as a, b, c and c’, as well as the significance level from the confidence interval of the indirect effect (a and b), it was observed that the mediator effect (indirect effect) between childhood HS and total body aBMD (β = 0.0025; 95% CI = 0.0005–0.0048), upper limbs aBMD (β = 0.0040; 95% CI = 0.0017–0.0069) was significant. Adulthood HS mediated approximately 52% of the relationship between childhood HS and total aBMD and 83% of the relationship between childhood HS and aBMD of the upper limbs (Fig. 2). No mediator effect (indirect effect) was observed between childhood sit-ups with lumbar spine aBMD.

## Discussion

The aim of the present study was to verify the relationship between MFI in childhood and aBMD of the different regions of interest in adulthood and to verify whether this relationship is mediated by performance in MFI in adulthood. The main finding of this study is that adulthood HS plays a mediating role in the relationship between childhood HS and total and upper limbs aBMD, pointing out that muscle fitness in childhood may be important aBMD determinants in adulthood, especially when muscle fitness performance is maintained in the transition to adulthood. Direct effect was observed between childhood sit-ups with lumbar spine aBMD.

The magnitude of the relationship between MFI in childhood with aBMD in adulthood found in the present study is low to moderate, according to results observed in literature [33, 34]. When considering sex, chronological age, childhood BMI z-score, and MFI in determining BHI, models explained from 6% (sit-ups test and lumbar spine aBMD) to 55% (HS and upper limbs aBMD). Although the magnitude of the relationship and the explanation of some models are discrete, it is important to point out some considerations. There is a wide variety of determinants that influence PBM [7], even if low, making these results worthy of attention. Lower correlation coefficients are generally found in longitudinal studies as the time interval between measurements increases, and in this study, this interval is on average 13 years, so discrete coefficients are assumed [33, 35]. Finally, small changes impact bone strength and postpone weakening that comes with osteoporosis [4, 7].

Few studies have attempted to observe these relationships longitudinally, mainly in childhood. Foley et al. [25] evaluated the relationship between physical fitness in childhood with adulthood BHI by calcaneal quantitative ultrasonography measurements, and found positive and significant association between standing long jump and BHI in females; however, when performance in the standing long jump in adulthood was controlled, this association was not significant. A few other longitudinal studies evaluated the relationship between BHI obtained by DXA in adulthood and MFI in adolescence [33, 34, 36, 37]. These studies are heterogeneous regarding the regions of interest measured by DXA and methods used such as MFI, but in general, they point to significant, positive, and low to moderate magnitude relationships.

Additionally, as conducted by Kemper et al. [34], this study controlled regression analyses with sex and also obtained significant relationships between childhood MFI and adulthood BHI. There is a need to control the variable sex in analyses involving muscle fitness and aBMD, due to hormonal aspects, body size [38] and preference for specific physical activities that are inherent to sex and may not reach thresholds that stimulate osteogenesis.

With regard to SLJ, muscle fitness indicator, showed no statistically significant relationship with aBMD for any of the regions of interest, although some studies have reported positive association [27, 41], others have reported negative association [40] and some found no association [41]. The aspect that may explain this result in motor tests involving body displacement to estimate muscle strength, is the body weight, which can play a key role in performance during the execution of tests, and those with weight values below average may perform better [40]. In addition, SLJ requires greater motor efficiency, so force generation with lower limb speed may be affected by poor motor coordination [40, 10].

On the other hand, relationships found between HS and upper limbs aBMD and sit-ups with lumbar spine aBMD can be understood through the mechanostatic theory, via interaction of bones with muscles [11]. The role of HS in childhood and adults with aBMD is worth highlighting. This relationship between HS and total body aBMD may be explained, in part, by the ability of the handgrip test to represent the individual’s overall strength level [42]. Furthermore, mechanical and biological stimuli can trigger generalized systemic endocrine effects, such as myokines in bone metabolism, which could explain the interaction of bone and muscle tissue even at anatomically distant sites [43].

With regard to mediation analysis, mediation of adulthood HS in relationships between childhood HS and total body and upper limbs aBMD and direct effect of childhood sit-ups on lumbar spine aBMD were found. This result allows concluding that muscle fitness performance in childhood provides higher adulthood aBMD, especially when higher muscle fitness performance is maintained in adulthood. Thus, the stimuli need to be constant in order to maintain the osteogenic effects. Furthermore, these relationships were longitudinally examined, while other studies have investigated mediator variables in similar relationships with cross-sectional design, such as the work by Torres-Costoso et al. [40] and Vicente-Rodríguez et al. [44]. A recent study found a mediator effect of muscle mass on the relationship between physical activity in child-hood and adolescence and bone parameters at the age of 17 years, highlighting the important effect of muscle fitness on BHI [45]. Studies have sought to analyze the mediator effect of muscular fitness on the association between other factors such as eating disorders [46], sports involvement [47], vitamin D [48], physical activity [49] and BHI.

Finally, direct relationship of sit-ups in childhood with aBMD of the lumbar spine in adulthood was observed, sit-ups test performance did not play a mediating role in the relationship of equivalents in childhood. The direct effect of childhood MFI on adulthood aBMD highlight the importance of improving muscle fitness from childhood.

The strength of this study is its longitudinal design, capable of partially inferring the causal relationship among variables analyzed. Follow-up studies can have a dropout effect, and in this analysis, they were negligible and not significant. Another potential was the assessment of bone variables and application of motor tests that analyze different body regions, allowing close and distant associations of anatomical specificities. Limitations include sample size, which made stratification by sex unfeasible, but allowed controlling sex in the analyses; the application of motor tests in the identification of muscle fitness, which suffers interference from other variables that could not be controlled; obtaining aBMD only at the adult moment and lack of control of other confounding variables, such as calcium, vitamin D intake, and practice of physical activity between childhood and adulthood. Future studies should invest in assessing and training muscular fitness to promote bone health at all stages of the life cycle, seeking to adequate the dose response needed to obtain advantages and to seek to maintain health benefits already obtained.

## Conclusion

It was possible to conclude that some MFI in childhood showed significative relationship with BHI in adulthood, such as between HS and total body and upper limbs aBMD, sit-ups test and lumbar spine aBMD. In addition, the sit-ups test in childhood had a direct effect on lumbar spine aBMD in adulthood, highlight the importance of improving muscle fitness from childhood. Adulthood HS mediated the relationship between childhood HS and total body and upper limbers aBMD, pointing out that muscle fitness in childhood may be an aBMD determinant in adulthood, especially when higher muscle fitness performance in adulthood is maintained.

## Data Availability

The datasets used and/or analyzed during the current study are available from the corresponding author on reasonable request.

## Abbreviations

MFI: Muscular fitness indicators
aBMD: Areal bone mineral density
BHI: Bone health indicators
HS: Handgrip strength
IE: Indirect effect
PBM: Peak bone mass
SLJ: Standing long jump test
DXA: Dual Energy X-Ray Absorptiometry
CMF z-Score: Childhood Muscular Fitness z-Score.
